# Supplementary material for: HIVprotI: an integrated web based platform for prediction and design of HIV proteins inhibitors
Source: J Cheminform. 2018 Mar 9;10:12. doi: 10.1186/s13321-018-0266-y (PMC5845081; doi:10.1186/s13321-018-0266-y)
Supplement: Supplementary file 2 — Additional file 2. Source code of HIVProtI web server. [file 13321_2018_266_MOESM2_ESM.zip › HIVprotI_Source-code/hivprotI/weka-3-6-6/doc/weka/clusterers/DensityBasedClusterer.html]

DensityBasedClusterer


---


|  |  |  |
| --- | --- | --- |
| **Overview** \| **Package** \| **Class** \| **Tree** \| **Deprecated** \| **Index** \| **Help** \| **Weka's home** | |  |
| **PREV CLASS**   **NEXT CLASS** | **FRAMES**    **NO FRAMES**     **All Classes** |  |
| SUMMARY: NESTED \| FIELD \| CONSTR \| METHOD | DETAIL: FIELD \| CONSTR \| METHOD |  |


---


## weka.clusterers Interface DensityBasedClusterer

**All Superinterfaces:**: Clusterer

**All Known Implementing Classes:**: AbstractDensityBasedClusterer, EM, MakeDensityBasedClusterer, RandomizableDensityBasedClusterer

---

``` public interface DensityBasedClusterer extends Clusterer ```

Interface for clusterers that can estimate the density for a given instance.
Implementations will typically extend AbstractDensityBasedClusterer.

**Version:**
:   $Revision: 1.9 $

**Author:**
:   Mark Hall (mhall@cs.waikato.ac.nz), Eibe Frank (eibe@cs.waikato.ac.nz)

---

| **Method Summary** | |
| --- | --- |
| `double[]` | `clusterPriors()`             Returns the prior probability of each cluster. |
| `double` | `logDensityForInstance(Instance instance)`             Computes the density for a given instance. |
| `double[]` | `logDensityPerClusterForInstance(Instance instance)`             Computes the log of the conditional density (per cluster) for a given instance. |
| `double[]` | `logJointDensitiesForInstance(Instance inst)`             Returns the logs of the joint densities for a given instance. |

**Methods inherited from interface weka.clusterers.Clusterer**

`buildClusterer, clusterInstance, distributionForInstance, getCapabilities, numberOfClusters`

**Method Detail**

### clusterPriors

```
double[] clusterPriors()
                       throws java.lang.Exception
```

:   Returns the prior probability of each cluster.

    :   **Returns:**: the prior probability for each cluster **Throws:**: `java.lang.Exception` - if priors could not be returned successfully

---


### logDensityPerClusterForInstance

```
double[] logDensityPerClusterForInstance(Instance instance)
                                         throws java.lang.Exception
```

:   Computes the log of the conditional density (per cluster) for a given instance.

    :   **Parameters:**: `instance` - the instance to compute the density for **Returns:**: an array containing the estimated densities **Throws:**: `java.lang.Exception` - if the density could not be computed successfully

---


### logDensityForInstance

```
double logDensityForInstance(Instance instance)
                             throws java.lang.Exception
```

:   Computes the density for a given instance.

    :   **Parameters:**: `instance` - the instance to compute the density for **Returns:**: the density. **Throws:**: `java.lang.Exception` - if the density could not be computed successfully

---


### logJointDensitiesForInstance

```
double[] logJointDensitiesForInstance(Instance inst)
                                      throws java.lang.Exception
```

:   Returns the logs of the joint densities for a given instance.

    :   **Parameters:**: `inst` - the instance **Returns:**: the array of values **Throws:**: `java.lang.Exception` - if values could not be computed


---


|  |  |  |
| --- | --- | --- |
| **Overview** \| **Package** \| **Class** \| **Tree** \| **Deprecated** \| **Index** \| **Help** \| **Weka's home** | |  |
| **PREV CLASS**   **NEXT CLASS** | **FRAMES**    **NO FRAMES**     **All Classes** |  |
| SUMMARY: NESTED \| FIELD \| CONSTR \| METHOD | DETAIL: FIELD \| CONSTR \| METHOD |  |


---
